# A Three Year Study on 14 VOCs at One Site in Rome: Levels, Seasonal Variations, Indoor/Outdoor Ratio and Temporal Trends

**DOI:** 10.3390/ijerph7103792

**Published:** 2010-10-22

**Authors:** Sergio Fuselli, Marco De Felice, Roberta Morlino, Luigi Turrio-Baldassarri

**Affiliations:** Igiene degli Ambienti di Vita, Dipartimento Ambiente e Connessa Prevenzione Primaria, Istituto Superiore di Sanità, Viale Regina Elena 299, 00161 Roma, Italia; E-Mails: sergio.fuselli@iss.it (S.F.); marco.defelice@iss.it (M.D.F.); roberta.morlino@iss.it (R.M.)

**Keywords:** VOCs, indoor air, outdoor air, benzene, automotive emissions

## Abstract

Fourteen volatile organic compounds (VOCs)—twelve hydrocarbons and two organochlorine compounds—were monitored both outdoors and indoors for three years at one site in Rome. Results showed that 118 out of 168 indoor seasonal mean values were higher than the corresponding outdoor concentrations. The most relevant source of outdoor hydrocarbons was automotive exhaust emissions. Due to the enforcement of various measures to protect health and the environment, outdoor levels of monoaromatic hydrocarbons decreased about ten fold over 15 years, and aliphatic hydrocarbons also decreased. With the decrease in these outdoor concentrations, indoor air sources are likely to be more relevant for indoor air exposures. Winter outdoor values for monoaromatic hydrocarbons were generally markedly higher than the summer ones. The gradual replacement of the current fleet of circulating cars with new cars complying with EURO 5 standards, further reducing hydrocarbon emissions, may possibly lead to an increase in the observed indoor/outdoor ratios. It is indeed more difficult to remove indoor sources, some of which are still unknown.

## 1. Introduction

The Istituto Superiore di Sanità (ISS) has a front garden site in which air pollutant sampling is performed. Monitoring began in the 1970s and is still continuing today for some compounds. For emerging pollutants, sampling began as a consequence of concerns that their toxicological properties induced health effects, and was performed in the frame of specific projects; in several cases monitoring continued indefinitely beyond project time frames.

Over time the number substances analyzed has increased: the first determinations included acid gases and particulate matter (PM) of 10 μm size (PM_10_), and eventually included monitoring of PM 2.5 μm (PM_2.5_), PM 1, metals present on PM [1], NO_2_, NO, NO_x_, CO, O_3_, particle number concentration (PNC) [2,3], as well as dioxin, polychlorinated biphenyls (PCBs) and polycyclic aromatic hydrocarbons (PAHs) [4–6]; the latter two classes of compounds were also monitored indoors [7].

This site appears to be one of most extensively studied in Europe for monitoring local air pollution. A monitoring program for outdoor volatile organic compounds (VOCs) started in 2003, and since 2007 the program was enlarged to include the same VOCs measured in an indoor site, chosen to be as close as possible to the outdoor one. Indoor exposures became increasingly relevant as it was shown that indoor air may contain substances not significantly present outdoors and that substances present outdoors often display higher concentrations indoors. This implies that, for these substances, indoor human exposure may be more relevant than the outdoor exposures, as most people spend more of their time indoors [8–10]; it should be noted that personal exposure is not a time-weighted average of indoor and outdoor concentrations [9].

The VOCs selected for the present study belong to two different categories: hydrocarbons and chlorinated hydrocarbons; the former are emitted outdoors, mainly by traffic, and indoors by glues, paint, fabric and plastic materials present in furniture and building materials, while the latter are present outdoors as industrial emissions, mainly from dry cleaning, and indoors from house cleaning products [11]. The choice of VOCs was aimed at outlining the influence of outdoor pollution on indoor air, with particular attention to automotive emissions. The choice of monoaromatic and light aliphatic hydrocarbons, as will be discussed later, might help determine a specific origin within automotive emissions.

The simultaneous and continued sampling of several indoor/outdoor sites and may help to trace the pollutant/emission sources, as well as the temporal and seasonal trends. Long-term monitoring, displaying temporal trends, may help to demonstrate the effectiveness of measures to reduce pollution. Finally, a data base of collected data may help to find correlations and set up models.

## 2. Methods

### 2.1. Site

The outdoor site is located on the roof of a cabin on the right side of the ISS main building central entrance. It is about 8 m from the curb of the street, a large road with a medium-high traffic intensity of about 25,000 vehicles per day [7], roughly at the road level (the ground floor of the right side of the building is lower than the street).

The indoor site is located in an office at the ground floor of the ISS main building, about 10 m to the left side of the outdoor site. The window facing the street is about 2 m lower than both the street and the outdoor site, approximately at the same height (2 m) from the ground as the outdoor site. It is the office closest to the outdoor site with no laboratories in the same corridor. The room has one door to the corridor and one window facing the street; air change is provided by the window which is often open in spring and fall; the window, a very old metal one, is far from being air tight. The door is always closed except for normal passages of the one employee working in 9 am to 4 pm shift. An air conditioner-heat pump, working on inner air, functions for most of the summer and on particularly cold winter days. The employee does not smoke and smoking is not allowed in the whole building.

### 2.2. Sampling

Sampling was performed with the Radiello^®^ [12] passive sampler; it consists of a cylindrical cartridge of activated charcoal. During sampling the cartridge was located inside a cylindrical micro-porous diffusion body made of polycarbonate and polyethylene mounted on a stand. The sampling was simultaneously performed at both the indoor and outdoor site for two weeks, to obtain two samples per month.

### 2.3. Analysis

After sampling, the cartridge was sealed in its container and stored at 4 °C until analysis. We have verified the statement of Radiello reference manual, section D4 [13], that a properly stored cartridge maintains its content unaltered for at least six months, by analyzing at different times replicate samples. Twelve (12) Radiello samplers were spiked by injecting 2 μL of a standard solution of CS_2_ containing the analytes into the bottom of the glass tube container of the sampler. The Radiello samplers were immediately inserted, sealed and left to stabilize for two days at 25 °C; then stored. The first set of four samplers was analyzed immediately after stabilization (t_0_), the second set of four samplers was analyzed after three months (t_90_) and the remaining set after six months (t_180_). The first two sets, roughly representing the extremes of our field conditions, were also used to determine the variability of the analytical method.

Analyses were performed at the end of each season on a batch of samples made of 12 single samples (two per month per site). For each batch two procedural blanks (one per site) were also analyzed. The results from each of the six samples collected at one site during the season were averaged to make a seasonal mean result.

Analytes were removed from the charcoal through desorption with 2 mL of carbon disulfide (Fluka, chromatography purity grade) under mechanical stirring for 30 min. Sampling and desorption were performed according to the Radiello standard operating procedures issued by the manufacturer [13].

Analyte identification and quantitation were performed with the external standard method by means of high resolution gas chromatography-low resolution mass spectrometry (HRGC-LRMS).

A GC Agilent Technologies mod. 6980 coupled with an Agilent Technologies MS mod. 5973N was used for the analysis with the following conditions: column: J&W DB-624 Durabond, 60 m long, 0.25 mm i.d., film thickness 1.4 μm. GC program: 35 °C for 3 min, up to 100 °C at 5 °C/min rate; 100 °C for 1 min; up to 240 °C at 10 °C/min; 240 °C for 3 min. MS conditions: IE 70 eV; full scan from 45 to 300 amu, 5 scan/sec.

The limits of quantitation, μg/m^3^, of the method, on a two week sampling period are reported in the first column of Table 1. They were determined with a procedure similar to the one used to study the variability of the method and the stability of the samples (injection of solution into the Radiello tube), with the difference that the different spiking solutions used were obtained by sequential 1:3 dilution of the starting solution. The LOQs so obtained are meant to be method limits, although the sampling is simulated; in fact, the procedure adopted allows determination of the lowest quantifiable concentration of sampled analyte. The limit of quantitation was established at a signal-to-noise (S/N) ratio = 5 (calculated from the last quantifiable analyte signal); the analytical procedure used implies that the difference between detection and quantification limit lies mainly in the S/N ratio. The S/N ratio adopted is on the lower side of what is accepted for a quantitation limit, while S/N = 3 generally defines the limit of detection [14]. No quantifiable analyte signal was ever detected in the blanks.

## 3. Results

The variability of the analytical method, different for each compound, varied from a minimum coefficients of variation (CV) of 6.2% for toluene to a maximum of 14.4% for 2-methylcyclopentane. The median CV value over the 14 VOCs was 7.72%.

Making the mean amount of each analyte in the (t_0_) set equal to 1, the (t_90_) mean values varied between 1.02 and 0.95 and the (t_180_) mean values between 1.03 and 0.93 (*n*-hexane). The stability of the samples under our storage conditions was thus verified.

In Table 1 the annual mean concentrations for the outdoor site measured in the years 2007–2009 are reported, together with their CVs. In Table 2 the results of the indoor site are reported.

The CVs are generally high for both indoor and outdoor sites, indicating wide variations in concentrations over the year. Within each year, however, the CVs of each season are generally lower (data not reported). Variations in indoor concentrations do not seem to differ greatly from the outdoor concentrations. Differences in the mean concentration of the various analytes over the three years should not be read as indicators of a time trend as the period of time is too short, the CVs too high and the differences too little to be significant.

In the Tables 3–5, we reported the results in terms of the ratio of the indoor/outdoor (I/O) concentration for each season and in terms of counts of the I/O values > 1 and counts of I/O values < 1 and, finally, in terms of the ratio of the concentrations in the extreme seasons (winter/summer, when the ventilation of the indoor site is respectively minimum and maximum and the occurrence of thermal inversion is, respectively, maximum and minimum).

In Table 3, the VOC I/O ratios are reported for each season of 2007, along with the number of values of seasonal I/O ratio higher than one and the ones lower than one, along with both indoor and outdoor results for winter/summer ratios. Table 4 and Table 5 report in the same order of Table 3 the data for the years 2008 and 2009, respectively.

## 4. Discussion

Considering the I/O ratios of the various VOCs, it emerges that 118 out of the 168 overall seasonal I/O ratios are higher than 1, which means for the 14 analytes determined in this study, indoor values are generally higher than outdoor values. Among our analytes there are compounds which are more concentrated indoors, compounds which are more concentrated outdoors, and compounds roughly at the same levels in both environments. The overall result obtained depends largely on the choice of the analytes. Our selection included 12 hydrocarbons, both aromatic and aliphatic, with 6 to 9 carbon atoms and two chlorinated hydrocarbons. The main outdoor source of the aliphatic hydrocarbons are both gasoline and gasoline-fueled vehicles; diesel fuel is a distillate [15] not containing compounds boiling under about 220 °C (the higher boiling compound in our list is *o*-xylene, boiling at 144 °C) but both gasoline and diesel vehicles can generate and emit monoaromatics during combustion [16,17]. The indoor sources of both kinds of hydrocarbons are solvents contained in glues, paints and synthetic plastics or released from materials such as the polyurethane foam, which can release previously accumulated VOCs. The urban outdoor sources of tetrachloroethylene are dry cleaning emissions, and the indoor sources are personal and housekeeping products not present in the office, as our data confirm. The presence of chloroform is unexpected and its source is unclear as most indoor sources are related to water, not present in the room. Among our analytes, concentrations of benzene and *n*-hexane were always higher indoors than outdoors (12 values higher than 1 out of 12 seasonal ratios I/O: 12/12); 2-methylhexane, methylcyclopentane and cyclohexane almost always (11/12), *n*-heptane, methylcyclohexane, toluene and chloroform very frequently (10/12). Only tetrachloroethylene showed frequent had higher levels (10/12) outdoors than indoors.

We found good correlations among all the series of twelve seasonal outdoor concentrations of the monoaromatic hydrocarbons: each monoaromatic compound is correlated with all the others. The data are presented in Table 6.

As demonstrated by Monod *et al.* [15], the correlation of both *m*+*p*-xylenes and ethylbenzene with toluene and benzene indicates that automotive combustion more than fuel evaporation is the main source of the outdoor pollution.

A correlation of the aliphatic hydrocarbons between each other for outdoor concentration does not exist in most cases; there are however some correlation coefficients that indicate that some hydrocarbons may have the same origin: 2-methylhexane and *n*-heptane, for instance. The higher values of the indoor concentrations indicate that for most of the hydrocarbons considered indoor sources add to the contamination brought inside by external air. However, from the I/O ratio or tetrachloroethylene it is reasonable to assume that all of the indoor load is brought inside by external air.

As stated previously, it is not possible to infer long term time trends from our data. It is however possible to extract some information by comparing the present data with literature data. Regular monitoring data are available for benzene, while less systematic data can be found on other hydrocarbons.

A comparison can be made with the official regional data of Rome monitoring, carried out by the ARPA Lazio (the regional agency for environmental protection), where sampling is performed daily at four outdoor stations, two of which have been at the same sites since 2003. One is located inside a wide urban park (villa Ada), and the other site is downtown on the side of a medium-high intensity traffic street (Magna Grecia street). The benzene annual means at these two stations both show a regularly decreasing trend for the 2003–2009 period [18–23], with the park site decreasing from to 2.5 to 1.5 μg/m^3^ and the downtown site decreasing from 5.4 to 2.7 μg/m^3^. So there is a good agreement between our outdoor annual means for benzene and the data from ARPA. The decreasing trend is more evident if older data are considered: in 1994 [24] a Rome street background level of 29 μg/m^3^ (mean of 12 sites, four seasonal replicates each) was measured for benzene, 93 μg/m^3^ for toluene, 106 μg/m^3^ for the sum of the three xylenes and 22 μg/m^3^ for ethylbenzene.

Brocco *et al.* [25] reported the following data (μg/m^3^) for hydrocarbon concentrations measured in Rome in the years 1992–1993 in two very central, high intensity traffic sites: benzene 35; toluene 100; *m*+*p* xylene 55; *o*-xylene 25; ethylbenzene 18; 1,2,4-trimethylbenzene 21; *n*-hexane 16; methylcyclopentane 13; *n*-heptane 10.

The two sets of historical data from the 1990s made almost contemporaneous measurements and they are in good agreement with each other, considering the slight differences in both time of sampling and traffic intensity. The values reported were not uncommon in those days; nor was it uncommon for the indoor values to be slightly lower than the corresponding outdoor values but also much higher than the outdoor values we measure nowadays. A study on indoor and outdoor benzene concentration in European cities for the 1996–2000 period [26] reported high values for Mediterranean cities and much lower values for northern cities. In Athens both outdoor and indoor benzene mean values were close to 8 μg/m^3^ and in Milan outdoor mean was about 12 μg/m^3^ with an indoor value of about 9 μg/m^3^. Another study from the same era (1997–1998) [27] in Athens reported an outdoor mean value of 18 μg/m^3^ and indoor values close to 9 μg/m^3^.

In contrast, when outdoor levels decrease, indoor sources appear to contribute to the outdoor load brought in by external air, making the indoor atmosphere more contaminated than outdoor air, as shown by several studies including those by Lai *et al.* [26] and Jia *et al.* [28].

Comparing the data from 1994 [24] to data from our present study, we see a decrease in outdoor monoaromatic hydrocarbons of about one order of magnitude in Rome streets over 15 years (1994–2009). If we compare the aliphatic hydrocarbons reported in Brocco *et al.* [25] with the present ones, the decrease is of a smaller magnitude but still noticeable and deserves comment. Several events happened in this period contributing to the reduction of pollution in general and of benzene in particular:

- the decrease of the maximum level of benzene in gasoline from 5 to 1%;
- the gradual replacement of circulating vehicles not equipped with three way catalyst with new low emission cars, also equipped with devices to prevent evaporative losses;
- the increased number of refilling stations equipped with the closed loop vapour system;
- the progressive elimination of two-stroke engines in motorcycles (two-stroke engines emit great amounts of unburned gasoline, and used to be very popular in Rome).

These events affected mostly gasoline and gasoline-fueled-vehicles. As previously mentioned, diesel vehicles emit monoaromatic compounds as combustion products, and in the same period emission standards of hydrocarbons for new diesel passenger cars also became more strictly regulated, passing from limit of the EURO 1 (1992, NO_x_ + HC = 0.97 g/km) to Euro 5 (2009, NO_x_ + HC = 0.23 g/km).

Table 7 reports the correlations between the indoor and outdoor concentrations of each compound. For most of the hydrocarbons the correlation is very poor; the correlation is stronger for those compounds, such as toluene and *m+p* xylene, whose outdoor concentration contributes most of the indoor concentration.

It is reasonable to think that a significant portion of the indoor concentrations of these compounds comes from outdoor air with little or no contribution from inside. For the other compounds, positive values of the coefficient indicate that outdoor air is only one component of the indoor concentration.

On the other hand, from the data presented, it is evident that indoor hydrocarbons levels are influenced by outdoor air, particularly the monoaromatic hydrocarbons. As previously stated, there are additional sources indoors that combine with the outdoor load to make indoor levels of many of the hydrocarbons analyzed higher than the outdoor levels; not all of these sources are known and new ones are still being found [28].

Most of the circulating vehicles during our sampling were likely to be EURO 2 and EURO 3 vehicles. However, also EURO 1 and pre-EURO vehicles were still circulating; these vehicles, and in particular non catalytic pre-EURO vehicles, are strong emitters and even a small number of them can make a significant contribution to outdoors emissions. These vehicles are gradually being replaced. Considering this fact and considering also that the emission standards for hydrocarbons decreased roughly threefold from EURO 2 to EURO 5 (enforced in 2009) for gasoline and diesel passenger cars, it seems reasonable to expect that future ongoing monitoring will show a decrease in outdoor concentrations of all hydrocarbons from automotive emissions. As a consequence, the indoor/outdoor ratio of these hydrocarbons will increase as there appear to be numerous indoor sources, some of which are still unidentified, and therefore more difficult to remove.

A final comment on the seasonality trend from our data, as indicated by the winter/summer ratios (Tables 3–5). Most of the ratios show values around 1 for both indoor and outdoor concentrations, showing the lack of a clear seasonality, except for the monoaromatic hydrocarbons, whose outdoor concentrations are generally significantly higher in winter than in summer. Consequently, the indoor winter/summer ratios of the same hydrocarbons are also higher than 1. This is probably due to the lower mobility of air in the wintertime due to thermal inversion and to the longer persistence of the compounds due to reduced photochemical degradation.

## Figures and Tables

**Table 1.** Yearly mean values (μg/m^3^) and CV% of outdoor air pollutants in the 2007–2009 period and their limit of quantitation (LOQ, μg/m^3^).

**LOQ**	**VOC**	**2007**		**2008**		**2009**	

		**Mean**	**CV%**	**Mean**	**CV%**	**Mean**	**CV%**
0.075	*n*-Hexane	5.3	14.7	5.2	32.2	6.1	24.8
0.014	Methylcyclopentane	2.4	19.3	2.4	36.1	2.2	22.2
0.033	Chloroform	0.3	28.7	0.3	33.7	0.2	49.8
0.015	2-Methylhexane	2.3	22.2	2.2	29.1	1.6	38.5
0.018	Cyclohexane	2.8	37.5	2.4	31.9	1.9	38.7
0.031	Benzene	2.7	44.1	3.0	39.8	2.7	46.1
0.086	*n*-Heptane	2.6	29.6	2.4	27.4	1.5	48.3
0.015	Methylcyclohexane	0.7	41.9	0.7	35.5	0.4	55.0
0.013	Toluene	10.8	36.1	10.2	33.4	9.4	32.8
0.017	Tetrachloroethylene	1.6	29.6	1.7	27.7	1.7	30.1
0.015	Ethylbenzene	1.4	33.8	1.4	36.6	1.2	39.6
0.014	*m*- *p*-Xylene	2.5	42.4	2.5	36.5	1.8	60.3
0.015	*o*-Xylene	1.6	36.3	1.8	32.6	1.4	38.4
0.020	1,2,4-Trimethylbenzene	2.2	44.8	2.3	38.6	2.0	76.5

**Table 2.** Yearly mean values (μg/m^3^) and CV% of Indoor air pollutants in the 2007–2009 period.

**VOC**	**2007**		**2008**		**2009**	

	**Mean**	**CV%**	**Mean**	**CV%**	**Mean**	**CV%**
*n*-Hexane	9.8	35.2	10.3	37.2	9.1	34.1
Methylcyclopentane	3.6	46.5	2.7	53.0	2.5	24.6
Chloroform	1.1	78.5	1.8	81.3	1.5	40.9
2-Methylhexane	3.2	38.3	4.0	43.1	3.2	20.7
Cyclohexane	3.4	47.7	3.2	83.9	2.5	43.0
Benzene	5.5	37.6	5.1	39.5	3.3	36.7
*n*-Heptane	3.6	44.5	4.2	41.1	4.2	13.8
Methylcyclohexane	1.3	61.7	1.1	48.9	1.0	38.5
Toluene	13.2	35.0	13.7	36.8	11.3	26.0
Tetrachloroethylene	1.7	72.4	1.3	52.6	1.2	54.2
Ethylbenzene	1.4	34.8	1.6	28.4	1.1	39.6
*m*- *p*-Xylene	2.2	40.1	2.1	40.0	1.6	46.3
*o*-Xylene	1.6	35.6	1.6	37.0	1.2	37.1
1,2,4-Trimethylbenzene	1.8	36.3	2.0	41.1	1.8	35.4

**Table 3.** Seasonal indoor/outdoor ratio during the year 2007, count of the I/O values higher or lower than 1 and winter/summer ratio.

**VOC**	**I/O**				Values > 1*	Values < 1**	**Indoor W/Su**	**Outdoor W/Su**
	**Winter**	**Spring**	**Summer**	**Fall**				
*n*-Hexane	1.6	2.0	1.9	1.9	4	0	0.8	0.9
Methylcyclopentane	1.1	1.2	1.5	2.0	4	0	0.7	0.9
Chloroform	0.7	0.8	6.0	6.6	2	2	0.2	1.2
2-Methylhexane	1.0	1.1	2.0	1.5	3	1	0.6	1.1
Cyclohexane	1.4	1.1	1.1	1.2	4	0	1.0	0.8
Benzene	1.4	1.8	3.3	2.5	4	0	1.2	2.8
*n*-Heptane	0.8	1.1	2.1	1.7	3	1	0.6	1.4
Methylcyclohexane	1.5	0.7	2.2	3.0	3	1	0.5	0.7
Toluene	0.9	1.4	1.5	1.3	3	1	1.2	2.0
Tetrachloroethylene	0.9	1.7	0.6	1.0	1	3	1.9	1.4
Ethylbenzene	0.6	0.9	1.5	1.1	2	2	0.8	1.9
*m*- *p*-Xylene	0.6	0.8	1.3	1.0	2	2	1.0	2.3
*o*-Xylene	0.6	1.0	1.5	1.0	3	1	0.9	2.1
1,2,4-Trimethylbenzene	0.6	1.2	1.3	0.7	2	2	1.1	2.3

Note: I/O = ratio of Indoor over Outdoor; W/Su = ratio of Winter over Summer;

* = overall 40.

** = overall 16.

**Table 4.** Seasonal indoor/outdoor ratio during the year 2008, count of the I/O values higher or lower than 1 and winter/summer ratio.

**VOC**	**I/O**				Values > 1*	Values < 1**	**Indoor W/Su**	**Outdoor W/Su**
	**Winter**	**Spring**	**Summer**	**Fall**				
*n*-Hexane	2.2	2.0	2.5	1.6	4	0	0.9	1.1
Methylcyclopentane	1.7	0.4	1.1	1.3	3	1	0.9	0.6
Chloroform	4.9	3.3	9.4	5.1	4	0	0.4	0.8
2-Methylhexane	2.2	1.2	1.9	1.9	4	0	0.9	0.8
Cyclohexane	3.7	0.3	1.1	1.2	3	1	1.8	0.6
Benzene	2.3	1.6	2.7	1.1	4	0	2.0	2.3
*n*-Heptane	1.6	1.0	2.0	2.3	3	1	0.8	1.0
Methylcyclohexane	1.9	0.8	1.8	1.7	3	1	0.7	0.6
Toluene	1.4	1.4	1.4	1.2	4	0	1.7	1.6
Tetrachloroethylene	0.8	0.6	0.7	1.0	1	3	1.3	1.1
Ethylbenzene	1.0	1.6	1.4	0.9	2	2	1.4	2.0
*m*- *p*-Xylene	0.7	0.8	0.9	0.9	0	4	1.5	1.9
*o*-Xylene	0.6	0.9	1.0	1.0	2	2	1.0	1.8
1,2,4-Trimethylbenzene	0.8	0.6	1.3	0.9	1	3	1.2	2.0

Note: I/O = ratio of Indoor over Outdoor; W/Su = ratio of Winter over Summer;

* = overall 38.

** = overall 18.

**Table 5.** Seasonal indoor/outdoor ratio during the year 2009, count of the I/O values higher or lower than 1 and winter/summer ratio.

**VOC**	**I/O**				Values > 1*	Values < 1**	**Indoor W/Su**	**Outdoor W/Su**
	**Winter**	**Spring**	**Summer**	**Fall**				
*n*-Hexane	1.5	1.5	1.4	1.6	4	0	0.9	0.9
Methylcyclopentane	1.3	1.2	1.0	1.2	4	0	1.1	0.9
Chloroform	7.4	5.2	5.5	7.8	4	0	2.0	1.5
2-Methylhexane	1.9	1.8	1.9	2.8	4	0	1.3	1.3
Cyclohexane	1.4	1.4	1.0	1.7	4	0	1.1	0.8
Benzene	1.1	1.6	1.5	1.1	4	0	1.9	2.4
*n*-Heptane	1.9	2.5	3.1	4.0	4	0	1.0	1.6
Methylcyclohexane	2.3	1.7	1.8	4.3	4	0	1.2	1.0
Toluene	1.2	1.3	0.9	1.4	3	1	1.9	1.5
Tetrachloroethylene	0.7	0.9	0.4	0.8	0	4	2.1	1.3
Ethylbenzene	0.9	1.1	0.9	0.8	1	3	1.5	1.6
*m*- *p*-Xylene	0.9	1.1	0.8	0.9	1	3	2.5	2.3
*o*-Xylene	0.9	1.0	0.8	0.8	1	3	1.7	1.6
1,2,4-Trimethylbenzene	0.8	1.6	1.4	0.7	2	2	1.2	2.2

Note: I/O = ratio of Indoor over Outdoor; W/Su = ratio of Winter over Summer;

* = overall 40.

** = overall 16.

**Table 6.** Correlations between the twelve seasonal outdoor values in the 2007–2009 period of the monoaromatic and aliphatic compounds.

	
**Aromatic compounds**		**r^2^**	**Aliphatic compounds**		**r^2^**
Benzene	Toluene	0.86	*n*-Hexane	Methylcyclopentane	0.41
Benzene	Ethylbenzene	0.90	*n*-Hexane	2-Methylhexane	0.14
Benzene	*m*- *p*-Xylene	0.81	*n*-Hexane	Cyclohexane	0.25
Benzene	*o*- Xylene	0.88	*n*-Hexane	*n*-Heptane	−0.20
Benzene	1,2,4-TMB	0.80	*n*-Hexane	Methylcyclohexane	0.04
Toluene	Ethylbenzene	0.87	Methylcyclopentane	2-Methylhexane	0.66
Toluene	*m*- *p*-Xylene	0.92	Methylcyclopentane	Cyclohexane	0.72
Toluene	*o*- Xylene	0.83	Methylcyclopentane	*n*-Heptane	0.33
Toluene	1,2,4-TMB	0.62	Methylcyclopentane	Methylcyclohexane	0.58
Ethylbenzene	*m*- *p*-Xylene	0.88	2-Methylhexane	Cyclohexane	0.81
Ethylbenzene	*o*-Xylene	0.93	2-Methylhexane	*n*-Heptane	0.88
Ethylbenzene	1,2,4-TMB	0.82	2-Methylhexane	Methylcyclohexane	0.77
*m*- *p*-Xylene	*o*- Xylene	0.91	Cyclohexane	*n*-Heptane	0.51
*m*- *p*-Xylene	1,2,4-TMB	0.67	Cyclohexane	Methylcyclohexane	0.83
*o*-Xylene	1,2,4-TMB	0.88	*n*-Heptane	Methylcyclohexane	0.53
	

Note: 1,2,4-TMB = 1,2,4-Trimethylbenzene

**Table 7.** Correlations indoor *versus* outdoor values in the 2007–2009 period of the monoaromatic and aliphatic compounds.

	
**Aromatic compounds**	**r^2^**	**Aliphatic compounds**	**r^2^**
	
Benzene	0.48	*n*-Hexane	0.35
Toluene	0.78	Methylcyclopentane	0.64
Ethylbenzene	0.43	2-Methylhexane	0.42
*m*-*p*-Xylene	0.80	Cyclohexane	0.13
*o*-Xylene	0.55	*n*-Heptane	−0.02
1,2,4-Trimethylbenzene	0.59	Methylcyclohexane	0.24
	
