# Correction: Fuselli, *et al.* A Three Year Study on 14 VOCs at One Site in Rome: Levels, Seasonal Variations, Indoor / Outdoor Ratio and Temporal Trends. *Int. J. Environ. Res. Public Health* 2010, *7*, 3792–3803

**Published:** 2010-10-22

**Authors:** Sergio Fuselli, Marco De Felice, Roberta Morlino, Luigi Turrio-Baldassarri

**Affiliations:** Igiene degli Ambienti di Vita, Dipartimento Ambiente e Connessa Prevenzione Primaria, Istituto Superiore di Sanità, Viale Regina Elena 299, 00161 Roma, Italia; E-Mails: sergio.fuselli@iss.it (S.F.); marco.defelice@iss.it (M.D.F.); roberta.morlino@iss.it (R.M.)

In the reference 27 of our paper published in International Journal of Environmental Research and Public Health 2010 [1], four authors instead of three are listed; it reads:

Chatzis, C.; Evangelos, C.; Alexopoulos, T.; Linos, A. Indoor and outdoor personal exposure to benzene in Athens, Greece. *Sci. Total Environ.* **2005**, *349*, 72–80.

The correct form is:

Chatzis, C.; Alexopoulos, E.C.; Linos, A. Indoor and outdoor personal exposure to benzene in Athens, Greece. *Sci. Total Environ.* **2005**, *349*, 72–80.
